# The role of ferroptosis in vascular endothelial cells and its role in the pathogenesis of cervical spondylosis of vertebral artery type: a comprehensive review

**DOI:** 10.1590/1414-431X2026e15287

**Published:** 2026-04-17

**Authors:** Xiaoyi Fan, Sisi Xu, Huiquan Gao

**Affiliations:** 1 Department of Rehabilitation Medicine, Weihaiwei People's Hospital, Weihai, Shandong, China; 2 Department of Radiotherapy, Yantai Yuhuangding Hospital, Yantai, Shandong, China

**Keywords:** Ferroptosis, Vascular endothelial cells, Cervical spondylosis of vertebral artery type, Pathogenesis

## Abstract

Ferroptosis is an iron-dependent programmed cell death characterized by lipid peroxidation. Ferroptosis plays a key role in the dysfunction of vascular endothelial cells (VECs), which may promote pathological vascular damage through oxidative stress, inflammatory response, and barrier integrity destruction. The pathogenesis of vertebral artery (VA) type cervical spondylosis (CSA) is complex. Cervical degeneration and other factors can induce ferroptosis of VA VECs. This review systematically reviews the cutting-edge research results on the biological characteristics of ferroptosis, analyzes its molecular mechanism in the regulation of VECs function, and systematically discusses its internal relationship with CSA pathogenesis. This study provides a new perspective on the pathogenesis of CSA, as well as a new theoretical basis and potential intervention targets for developing targeted treatment strategies and disease prevention.

## Introduction

Cervical spondylosis of vertebral artery type (CSA) is a common clinical subtype of cervical spondylosis, and its pathophysiological mechanism involves the synergistic effect of multi-dimensional pathogenic factors. The main manifestations are vertigo, nausea, vomiting, and a series of symptoms caused by vertebrobasilar insufficiency, which seriously affect the quality of life of patients (1,2). The pathogenesis of this disease is complex, mainly involving cervical spine degeneration, vertebral artery (VA) compression, vascular wall lesions, hemodynamic changes, and other factors (3). In recent years, with the deepening of the research on the mechanism of cell death, ferroptosis, as a new type of programmed cell death, has gradually become a research hotspot because of its unique regulatory mechanism and biological characteristics. Ferroptosis plays an important role in the occurrence and development of a variety of diseases, including vascular diseases and nervous system diseases. As an important functional unit of the vascular wall, the integrity and functional status of vascular endothelial cells (VECs) directly determine the homeostasis and hemodynamic balance of the vascular system (4). Increasing evidence shows that ferroptosis plays an important role in the regulation of VECs (5). In this context, in-depth exploration of the regulatory mechanism of ferroptosis in VECs and its role in the pathogenesis of CSA has important theoretical and clinical significance for understanding CSA pathogenesis and identifying potential therapeutic targets.

## Biological characteristics of ferroptosis

### Definition of ferroptosis

Ferroptosis is an iron-dependent programmed cell death characterized by lipid peroxidation (6), with unique morphological, biochemical, and molecular biological characteristics. Morphological observations indicate that cells undergoing ferroptosis show destruction of cell membrane integrity and significant pyknotic changes in mitochondria. Specifically, membrane density is increased, mitochondrial volume is reduced, and mitochondrial cristae are reduced or disappear completely, while the nuclear morphology remains basically normal (7). Biochemically, ferroptosis is accompanied by massive accumulation of lipid peroxides and depletion of glutathione (GSH) (8). At the molecular level, the occurrence of ferroptosis is mainly regulated by glutathione peroxidase 4 (GPX4), iron metabolism-related proteins, and lipid metabolism-related proteins (9).

### Mechanisms regulating ferroptosis

**Figure 1:**
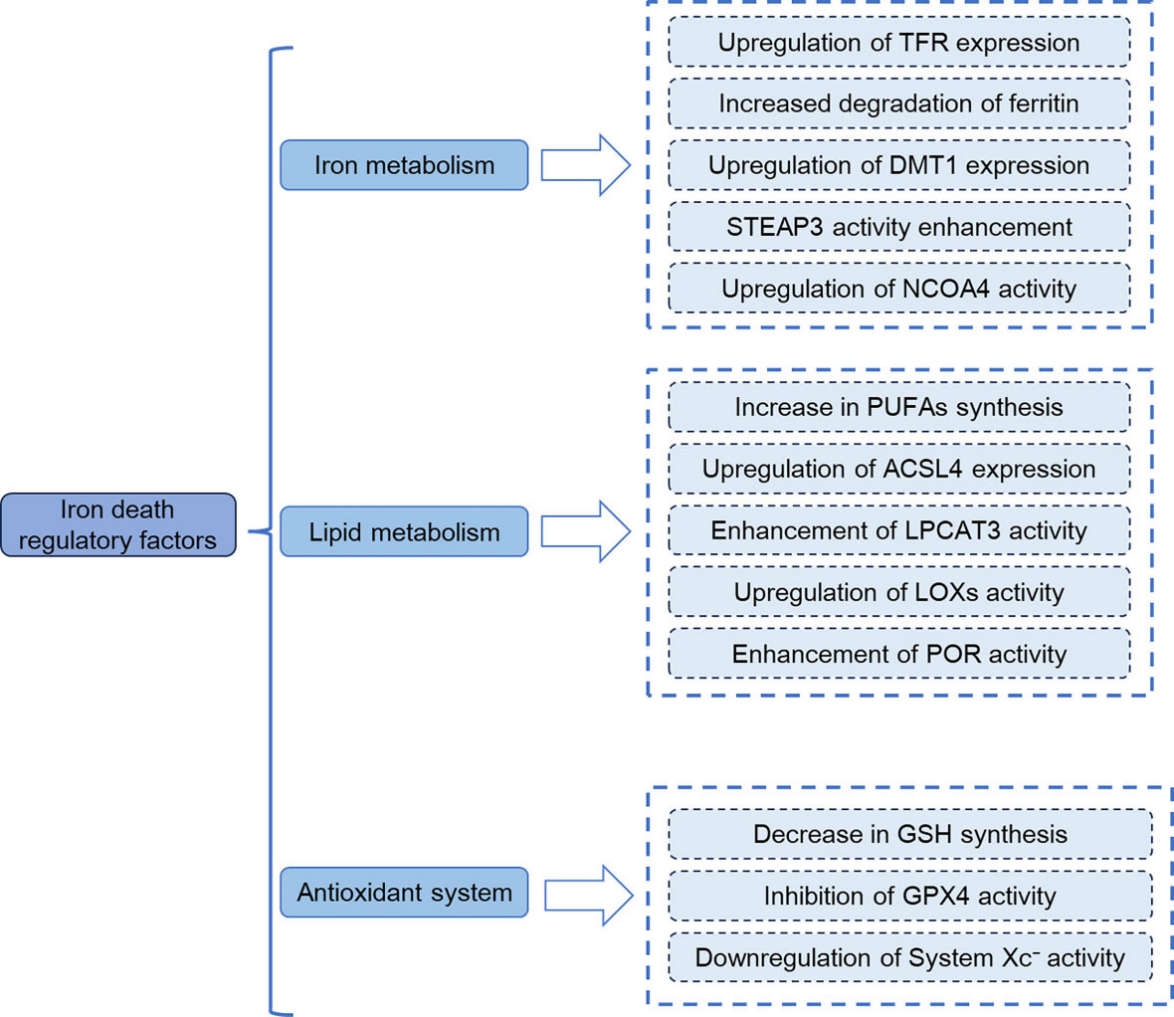
Schematic diagram of regulatory factors of ferroptosis. The regulatory mechanisms of ferroptosis mainly involve three aspects: iron metabolism, lipid metabolism, and the antioxidant system. TFR: transferrin receptor; DMT1: divalent metal transporter 1; STEAP3: six-transmembrane epithelial antigen of prostate 3; NCOA4: nuclear receptor coactivator 4; PUFAs: polyunsaturated fatty acids; ACSL4: Acyl-CoA synthetase long-chain family member 4; LPCAT3: lysophosphatidylcholine acyltransferase 3; LOXs: lipoxygenases; POR: cytochrome P450 oxidoreductase; GSH: glutathione; GPX4: glutathione peroxidase; system Xc^−^: cystine-glutamate antiporter.

As shown in Figure 1, regulatory mechanisms of ferroptosis mainly include three aspects: iron metabolism, lipid metabolism, and the antioxidant system.

Intracellular accumulation of ferrous ions (Fe^2+^) is a core prerequisite for the initiation of ferroptosis (10,11), and upregulation of divalent metal transporter 1 (DMT1) expression is one of the key mechanisms driving intracellular Fe^2+^ enrichment (12). The upregulation of transferrin receptor 1 (TfR1) can promote the intracellular transport of the transferrin (Tf)-Fe^3+^ complex. After Fe^3+^ is reduced to Fe^2+^ by six-transmembrane epithelial antigen of prostate 3 (STEAP3), DMT1 is required to transport Fe^2+^ from endosomes to the cytoplasmic labile iron pool (LIP). Additionally, the activation of nuclear receptor coactivator 4 (NCOA4)-mediated ferritinophagy induces the degradation of ferritin and the release of Fe^2+^. This process, together with DMT1-mediated iron transport, synergistically exacerbates Fe^2+^ accumulation. Excessive Fe^2+^ generates hydroxyl radicals (•OH) through the Fenton reaction, laying the foundation for the initiation of ferroptosis (13,14).

Lipid metabolism provides key substrates and toxic products for ferroptosis (15,16). First, acyl-CoA synthetase long-chain family member 4 (ACSL4) catalyzes the activation of polyunsaturated fatty acids (PUFAs) into PUFA-CoA. Subsequently, lysophosphatidylcholine acyltransferase 3 (LPCAT3) integrates PUFA-CoA into membrane phospholipids, forming oxidizable PUFA-modified phospholipids (PUFA-PLs) (17). In the following step, PUFA-PLs undergo peroxidation catalyzed by lipoxygenases (LOXs, e.g., ALOX15) or cytochrome P450 oxidoreductase (POR), generating phospholipid hydroperoxides (PLOOHs). These PLOOHs are further decomposed into toxic substances such as 4-hydroxynonenal (4-HNE), which disrupt the integrity of the cell membrane and drive the progression of ferroptosis (18-20).

Dysfunction of the antioxidant system is a direct inducer of ferroptosis, which primarily relies on the system Xc^−^-GSH-GPX4 axis (21). System Xc^−^ is composed of solute carrier family 7 member 11 (SLC7A11) and solute carrier family 3 member 2 (SLC3A2), and it mediates the uptake of extracellular cystine. After cystine is reduced to cysteine, GSH is synthesized. As a coenzyme of glutathione peroxidase 4 (GPX4), GSH provides the necessary substrate for GPX4 to scavenge lipid peroxides, thereby maintaining intracellular redox homeostasis (22). When the function of this axis is impaired - such as when GPX4 activity is inhibited or its expression is downregulated - lipid peroxides cannot be timely cleared and accumulate in large quantities, ultimately inducing ferroptosis (23-26).

In addition, other signaling pathways such as NRF2 (NF-E2-related factor 2) and p53 are also involved in the regulation of ferroptosis. NRF2 can enhance the antioxidant capacity of cells by activating the expression of downstream antioxidant genes, thereby inhibiting ferroptosis (27). p53 can promote or inhibit the ferroptosis by regulating the expression of genes related to iron metabolism and lipid metabolism (11).

### Pathological role of ferroptosis in VECs

VECs have a variety of important functions such as maintaining the integrity of the vascular wall, regulating vasomotor activity, participating in the balance between coagulation and fibrinolysis, and mediating inflammatory responses (28). The activation of the ferroptosis process can have a significant impact on the normal physiological function of VECs. On the one hand, ferroptosis leads to VECs injury, reduces the expression of intercellular junction proteins such as VE-cadherin, destroys the tight junction between cells, and increases vascular permeability (29,30). On the other hand, ferroptosis-induced lipid peroxidation can damage the membrane structure and function of VECs, affect the activities of ion channels, receptors and transporters on the membrane, and thereby interfere with the regulation of vasomotion and contraction by VECs (31,32). In addition, ferroptosis can also promote VECs to release inflammatory factors and chemokines, such as tumor necrosis factor-α (TNF-α) and interleukin-6 (IL-6), activate the inflammatory response, attract white blood cells to adhere to the surface of VECs, and further aggravate VEC damage (33).

### Association of ferroptosis with vascular disease

Ferroptosis plays an important role in the occurrence and development of a variety of diseases related to VEC injury. Oxidized low-density lipoprotein (ox-LDL) can induce VEC ferroptosis, promote inflammatory cell infiltration and lipid deposition, and accelerate the formation of atherosclerotic plaque in atherosclerosis (34,35). Studies have shown that inhibition of ferroptosis can attenuate the progression of atherosclerosis. For example, in a high-fat diet-induced ApoE−/− mouse model, ferroptosis inhibitor Ferrostatin-1 (Fer-1) significantly attenuated atherosclerosis by reducing iron accumulation and lipid peroxidation (36). Ferroptosis is closely related to the pathological processes of cerebrovascular diseases, such as intracerebral hemorrhage and subarachnoid hemorrhage. In diabetes-related cerebral ischemic injury, ferroptosis leads to the damage of brain microvascular endothelial cells through the inhibition of GPX4 activity and the activation of related signaling pathways (33). In diabetic vasculopathy, hyperglycemia can promote the ferroptosis of VECs by activating protein kinase C (PKC) signaling pathway, and then cause diabetic retinopathy, diabetic nephropathy, and other complications (37,38). In ischemia-reperfusion injury, reperfusion after ischemia leads to intracellular iron overload and increased reactive oxygen species (ROS) production, induces ferroptosis of VECs, and aggravates tissue damage (39). These studies indicate the important role of ferroptosis in vascular diseases, and modulation of the ferroptosis process may become a novel therapeutic strategy.

## Pathogenesis of CSA

CSA is a clinical syndrome of posterior circulation insufficiency caused by compression or spasm of the VA caused by cervical degenerative diseases. Its pathogenesis involves multiple links such as mechanical compression, hemodynamic abnormalities, endothelial injury, and ischemia/oxidative stress (1). The typical symptoms of CSA include episodic vertigo, visual disorders (amaurosis, blurred vision), headache, and cataplexy (40). In severe cases, transient ischemic attack (TIA) or cerebral infarction may occur (41).

Cervical degeneration is the pathological basis of the occurrence and development of CSA. With aging, cervical intervertebral discs, vertebral bodies, facet joints, and uncovertebral joints gradually show degenerative changes. The specific manifestations are disc herniation, vertebral bone hyperplasia, facet joint hypertrophy, and uncovertebral joint dysplasia. The above degenerative diseases can directly exert mechanical compression on the VA, resulting in the narrowing of the VA lumen diameter and change of hemodynamic parameters, leading to the insufficiency of the vertebrobasilar artery system (42,43).

In addition to mechanical compression, VA wall lesions and hemodynamic changes also play an important role in the pathogenesis of CSA. Long-term hemodynamic abnormalities, oxidative stress, inflammatory response, and other factors can lead to VEC damage in the VA, cause vascular wall inflammation, intimal hyperplasia, lipid deposition, and other pathological changes, and further aggravate VA stenosis (44). At the same time, VA stenosis can lead to accelerated and disordered local blood flow, forming vortex and turbulence, generating shear stress, further damaging VECs, and forming a vicious circle (43). In addition, vascular wall lesions can also affect the elasticity and relaxation function of blood vessels, resulting in decreased vascular regulation ability, which cannot meet the physiological needs of the body, thus aggravating the symptoms of vertebrobasilar insufficiency (45).

Ischemia and oxidative stress are key factors in the pathogenesis of CSA. After VA compression, blood flow is reduced, leading to ischemia, which in turn triggers oxidative stress. Oxidative stress can lead to apoptosis, mitochondrial dysfunction, and tissue damage (46). For example, in peripheral arterial disease (PAD), ischemia and oxidative stress together cause apoptosis and mitochondrial dysfunction in skeletal muscle cells (47).

## Potential association of ferroptosis with CSA

### Ferroptosis and VA VEC injury

**Figure 2:**
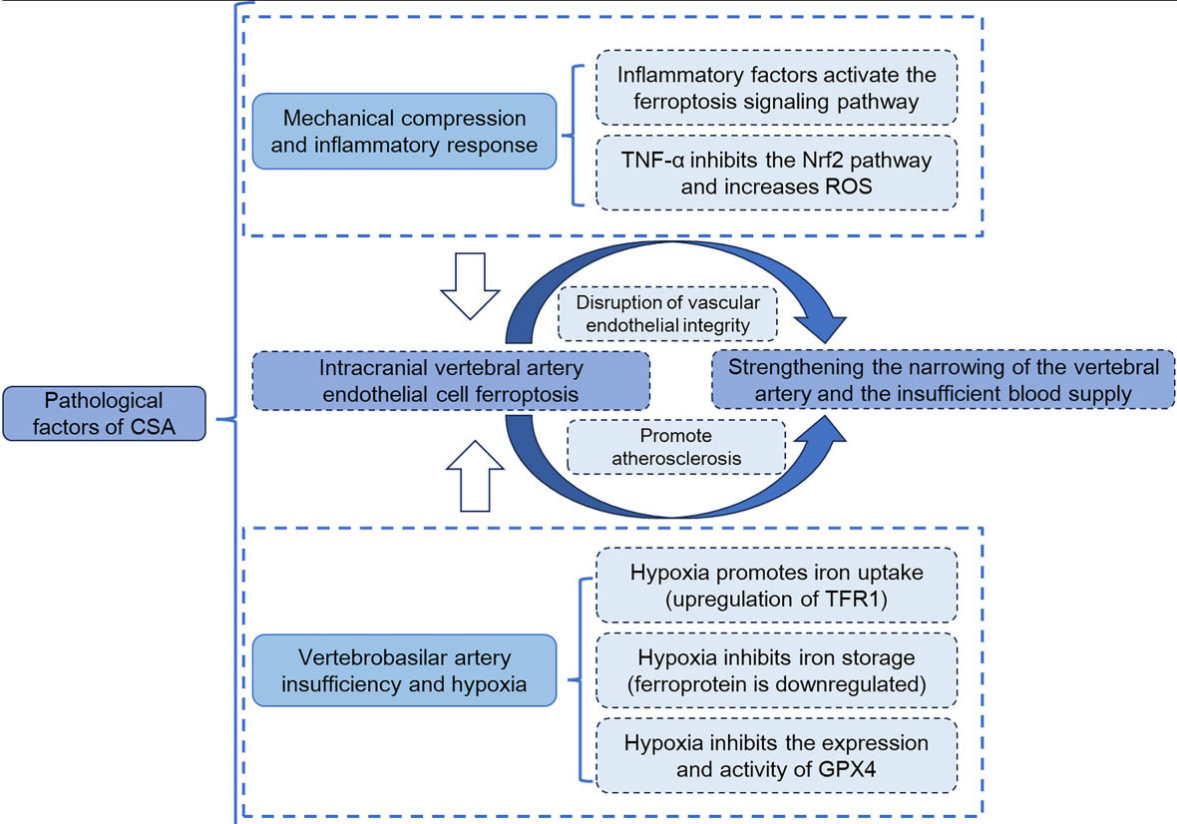
The mechanism of ferroptosis in the pathogenesis of CSA lesions. CSA's pathological factors (mechanical compression/inflammation, hypoperfusion/hypoxia) induce vertebral artery endothelial ferroptosis, forming a cycle that worsens CSA. CSA: cervical spondylotic arteriopathy; TNF-α: tumor necrosis factor-α; Nrf2: nuclear factor erythroid 2-related factor 2; ROS: reactive oxygen species; TFR1: transferrin receptor 1; GPX4: glutathione peroxidase 4.

In the pathogenesis of CSA, numerous factors, including mechanical compression, ischemia-hypoxia, and inflammatory responses, have been shown to induce ferroptosis in VECs in recent studies. A study published in 2021 revealed that mechanical compression can cause physical damage to VECs, leading to intracellular iron metabolism disorders (48). As a result, the accumulation of iron ions is promoted. Meanwhile, another paper suggests that compression can trigger oxidative stress responses due to local tissue ischemia-hypoxia (49). This process generates a large number of ROS, which in turn induce lipid peroxidation and activate the ferroptosis signaling pathway. Additionally, inflammatory factors such as TNF-α and IL-6 from the inflammatory response can also promote ferroptosis in VECs. They achieve this by regulating the expression of proteins related to iron metabolism, lipid metabolism, and the antioxidant system, as reported in a 2023 study (50). Ferroptosis-induced damage to VECs disrupts the integrity and function of the vascular endothelium. It increases vascular permeability and promotes the infiltration of inflammatory cells and thrombosis formation, as shown in multiple studies (51). These effects further exacerbate VA stenosis and vascular wall lesions, affecting the blood perfusion of the VA and participating in the pathological process of CSA. Figure 2 summarizes the key molecular events and pathogenic cycle underlying this association: CSA's pathological factors induce intracranial vertebral artery endothelial ferroptosis via specific pathways, which in turn aggravates vascular injury and perpetuates CSA progression.

### Effect of ferroptosis on VA hemodynamics

Ferroptosis-induced damage to VA VECs exerts a profound influence on VA hemodynamics. Studies have shown that when VECs are injured, the secretion of vasoactive substances, including nitric oxide (NO) and endothelin-1 (ET-1), is dysregulated, which disrupts the balance between vasodilatory and contractile mechanisms (52). NO is recognized for its vasodilatory properties, which effectively reduce vascular resistance, whereas ET-1 elicits vasoconstrictive responses (53). Cumulative evidence from recent studies has demonstrated that ferroptosis significantly attenuates the synthesis and release of NO by VECs while concurrently augmenting ET-1 secretion (54). This dysregulation culminates in vasoconstriction of the VA, luminal stenosis, elevated blood flow resistance, and decelerated blood flow velocity. Furthermore, as reported in a 2024 study, VECs injury compromises the integrity and smoothness of the vascular wall, precipitating blood flow disturbances characterized by the formation of eddies and turbulent flow patterns (55). These hemodynamic alterations exacerbate vertebra-basilar insufficiency, thereby contributing to the pathophysiology of CSA.

### Ferroptosis and inflammatory response in CSA

Ferroptosis is closely related to the inflammatory response in CSA. As mentioned above, several articles support that ferroptosis promotes the release of inflammatory factors and chemokines from VECs, thereby activating the inflammatory response (56,57). In CSA, the injury of VECs caused by ferroptosis triggers a local inflammatory response. Inflammatory cells such as monocytes and macrophages infiltrate the lesion site under the chemotaxis of inflammatory factors and release more inflammatory mediators, forming an inflammatory cascade reaction, as reported in a 2024 study (58). These inflammatory mediators not only further damage VECs and promote the occurrence of ferroptosis but also exacerbate the inflammatory response in the local tissues of the cervical spine (59). This results in pain and functional impairment of the muscles, ligaments, and other tissues around the cervical spine (60). At the same time, the inflammatory response can also affect the structure and function of the vascular wall of the VA, accelerating the progression of CSA, as indicated by multiple recent studies (61).

## Potential therapeutic strategies for targeting ferroptosis

In recent years, targeted ferroptosis has become a research hotspot in disease treatment. In the regulation of iron metabolism, reducing intracellular iron overload by iron chelating agents (such as deferoxamine) can inhibit the Fenton reaction and reduce the generation of ROS, thereby blocking the ferroptosis process (62). In addition, deferoxamine can also reduce the toxicity of iron by promoting the degradation of ferritin in lysosomes and transferring iron from inside to outside of cells (63,64). In cardiovascular diseases, iron chelators such as deferiprone (DFP) reduce myocardial injury and ischemia-reperfusion injury by regulating iron metabolism (65). In the regulation of lipid metabolism, inhibition of LOX activity or reduction of PUFAs supply can reduce the substrate of lipid peroxidation and hinder the occurrence of ferroptosis (66). Phosphatidylethanolamine binding protein 1 inhibitors can regulate lipid peroxidation and become a new therapeutic target (67,68). At the antioxidant system level, activation of nuclear factor erythroid 2-related factor 2 signaling pathway to promote the expression of downstream antioxidant genes, or exogenous supplementation of GSH and activation of GPX4 activity can enhance the antioxidant capacity of cells and resist ferroptosis (69-71). These strategies targeting ferroptosis provide new ideas for the treatment of CSA.

## Conclusions

As a new type of programmed cell death, ferroptosis plays an important role in the regulation of VECs and is closely related to the pathogenesis of CSA. Ferroptosis is involved in the pathological process of CSA by causing damage to VECs of VA, affecting VA hemodynamics and participating in inflammatory response. The detailed content is shown in Figure 2. However, current research on ferroptosis in CSA is still at an early stage, and many issues require further in-depth exploration. For instance, the specific mechanism of ferroptosis in different pathological stages of CSA is not yet clear; how to precisely regulate ferroptosis to achieve the goal of treating CSA; and whether there are other as-yet-unidentified signaling pathways related to ferroptosis involved in the pathogenesis of CSA. Future research should further explore the mechanism of ferroptosis in CSA, find effective targets for regulating ferroptosis, and provide new theoretical basis and treatment strategies for the prevention and treatment of CSA.

## Data Availability

The data used and analyzed can be obtained from the corresponding author upon a reasonable request.
